# Preparation and Characterization of a Novel Cassava Starch-Based Phosphorus Releasing Super-Absorbent Polymer, and Optimization of the Performance of Water Absorption and Phosphorus Release

**DOI:** 10.3390/polym15051233

**Published:** 2023-02-28

**Authors:** Wenbo Bai, Bingyi Ji, Liren Fan, Qin Peng, Qi Liu, Jiqing Song

**Affiliations:** 1 Institute of Environment and Sustainable Development in Agriculture, Chinese Academy of Agricultural Sciences, Beijing 100081, China; 2 Liaoning Province Modern Agricultural Production Base and Construction Engineering Center, Shenyang 110032, China; 3 Faculty of Materials Science and Chemistry, China University of Geosciences (Wuhan), Wuhan 300057, China

**Keywords:** super-absorbent polymer (SAP), water absorbency, swelling process, phosphorus release performance, structural characterization

## Abstract

To prepare a novel cassava starch-based phosphorus releasing super-absorbent polymer (CST-PRP-SAP), the single factor and orthogonal experiment were applied to analyze the effects of different reaction conditions on the absorption and phosphorus release capacities of CST-PRP-SAP samples. The structural and morphological characteristics of the cassava starch (CST), powdered rock phosphate (PRP), cassava starch-based super-absorbent polymer (CST-SAP) and CST-PRP-SAP samples were all compared with various technologies, such as the Fourier transform infrared spectroscopy and X-ray diffraction pattern, etc. The results showed that the CST-PRP-SAP samples had good performances of water retention and phosphorus release which were synthesized, such as the reaction temperature, starch content, P_2_O_5_ content, crosslinking agent, initiator, neutralization degree, and acrylamide content, which were 60 °C, 20% *w*/*w*, 10% *w*/*w*, 0.02% *w*/*w*, 0.6% *w*/*w*, 70% *w*/*w*, and 15% *w*/*w*, respectively. The water absorbency of CST-PRP-SAP was almost larger than that of the CST-SAP sample with a P_2_O_5_ content of 5.0% and 7.5%, and they all gradually decreased after three consecutive water absorption cycles. The CST-PRP-SAP sample could maintain about 50% of the initial water content after 24 h, even at the temperature of 40 °C. The swelling process of CST-PRP-SAP conformed to the non-Fickian diffusion, which was determined by the diffusion of water molecules and the relaxation process of polymer chain segments. The cumulative phosphorus release amount and release rate of the CST-PRP-SAP samples were increased with the increasing PRP content and the decreasing neutralization degree. After a 216 h immersion, the cumulative phosphorus release amount and release rate of the CST-PRP-SAP samples with different PRP contents were increased by 17.4 and 3.7 times, respectively. The rough surface of the CST-PRP-SAP sample after swelling was beneficial to the performance of water absorption and phosphorus release. The crystallization degree of PRP in the CST-PRP-SAP system was decreased and most of the PRP existed in the form of physical filling, and the available phosphorus content was increased to a certain extent. It was concluded that the CST-PRP-SAP synthesized in this study has excellent properties of continuous water absorption and retention with functions of promotion and the slow-release phosphorus.

## 1. Introduction

The super-absorbent polymer (SAP) is a kind of functional polymer material born in the 1960s. It has a high molecular weight and complex and diverse structural characteristics common to general polymer materials. It also contains a large number of strong water-absorbing groups with specific structures, which can quickly absorb water equivalent to hundreds or thousands of times its own weight, and has the function of repeated water absorption and slow release [1]_._ The application of SAP was considered as a chemical water-saving technology that could supply water to plants and improve soil structure by improving the root–soil interface environment of plants, and these characteristics made SAP increasingly become one of the rational technical measures for the development of water-saving agriculture in arid regions [2,3].

At present, the research on SAP in the agriculture and forestry field mainly focuses on the development and performance optimization of new composite functional SAP materials and products [4,5], influences of SAPs on soil physicochemical properties, crop growth and yield [6,7], interaction effects between SAPs and fertilizer [8,9], and the evaluation of comprehensive effects of SAPs applied in dryland agriculture [10,11]. A large number of studies have shown that SAPs can improve soil water holding capacity, increase soil infiltration, improve soil structure [7,12,13], enhance crop water and fertilizer utilization efficiency, and promote crop growth [14,15]. Most of the SAPs used in agriculture and forestry production are polyacrylamide polymers. Due to the relatively poor salt tolerance of SAPs, their applications would be affected by ions in soils or fertilizers, and the water retaining and soil improvement performances might be significantly weakened [16]. The application of compound fertilizer and SAPs in different soil layers has been used to reduce the influence of fertilizers on SAPs water-absorbing performance, but this might be bound to increase difficult fertilization operations and labor costs. In addition, the high cost of polymer SAP also greatly limits its widespread application in agriculture. With a new demand for efficient and sustainable agriculture, the development and application of functional new products using chitosan, starch, or other biomass resources and raw materials has gradually become an important direction for the development of this field [17].

Native starch has a high water-holding capacity but it is susceptible to environmental influences. However, when starch is grafted with a variety of vinyl monomers, such as acrylic amide (AM), acrylic acid (AA), acrylonitrile, methylacrylonitrile, and alkylmethacrylates, the application scope can be increased without sacrificing its biodegradable nature [18]. Cassava starch (CST) is among the most important starches in the industry, especially in countries such as India, Thailand, Indonesia, and Vietnam [19]. The amylopectin in the CST structure accounts for 83%, and it is more suitable for a grafting reaction with a vinyl monomer. Starch-based SAP that works by chelating organic compounds has emerged as a strong area of interest due to its obvious advantages in biodegradation and cost reduction compared with the polymer SAP. It has a wide application prospect in agricultural production because of its reproducibility that can be isolated from many natural substances such as wheat, corn, paddy, potato, and other agricultural products. Phosphorus is a major key nutrient for plants and affects several characteristics of plant growth. Though phosphorus, both in organic and inorganic forms, is abundant, due to its ability to form complexes with other soil constituents, it is not easily available for uptake by the plants [20]. Rock phosphate (RP) is a natural phosphorus source which can be an alternative to chemical fertilizers and can slowly release part of the fixed phosphorus to supply crops under certain environmental conditions [21]. For the research and development of low-cost functional SAPs application, there have been a few reports on single nutrient or micronutrient slow-release SAPs [22], new materials with functions of both nutrient slow-release and water retention [23], and the compound nutrient with long-acting substance [24].

Therefore, in order to develop a low cost, eco-friendly, and sustainable system where the ability of water absorption and retention, and supply of phosphorus to plants can be ensured, this study was undertaken to explore the possibility of increasing the phosphorus release capacity from low-grade RP. Optimization of the synthesis conditions and the performance of water absorption and phosphorus release by adding powdered rock phosphate (PRP) into the cassava starch-based super-absorbent polymer (CST-SAP) was the main objective of our study. The morphologies and structures of the PRP, CST, CST-SAP, and cassava starch-based phosphorus releasing super-absorbent polymer (CST-PRP-SAP) were all investigated and compared using Fourier transform infrared spectroscopy (FTIR), X-ray diffraction (XRD), and scanning electron microscopy (SEM). The adsorption capacity and adsorption kinetics of the CST-PRP-SAP were evaluated. Furthermore, the effects of the PRP and AM content, neutralization degree, crosslinking agent, and initiator amount on the abilities of water absorption and phosphorus release were all studied in detail.

## 2. Materials and Methods

### 2.1. Materials

The industrial low-grade RP powder used in the experiment was from Kaiyang, Guizhou Province, China. The main effective components were P_2_O_5_ 34.53%, CaO 49.13%, MgO 0.48%, CO_2_ 2.60%, F 2.70%, SiO_2_ 8.45%, Fe_2_O_3_ 0.55%, and Al_2_O_3_ 0.75%. The RP powder samples were pulverized by the vibrating disk mill (Retsch, RS200, Germany), and then the mineral powder samples with particle sizes less than 200 mesh (<74 μm) were selected by the standard criteria, dried and bagged, and prepared as PRP samples.

Commercial edible CST powder was obtained from Guangxi. The reactive monomers, AA and AM, were supplied by Tianjin Beichen Fangzheng Chemical Reagent Co., and Tianjin Kemiou Chemicals Factory Co., respectively. The crosslinking agent *N*,*N*-methylenebisacrylamide (*N*,*N*-MBA) was purchased from Tianjin Kemiou Chemicals Factory Co. The reaction initiators, ceric ammonium nitrate (CAN) and ammonium persulfate (APS), were obtained from Tianjin De’en Chemicals Factory Co. The reagents were of analytical grade. The solutions were prepared using distilled water. 

### 2.2. Preparation of CST-SAP

The CST-SAP samples were prepared by graft copolymerization of AA and AM onto a CST backbone in the presence of CAN and APS as the reaction initiators, followed by alkali saponification using a sodium hydroxide solution. The detailed procedure for the synthesis of CST-SAP has been previously reported [25]. The reaction conditions for the CST-SAPs were as follows: temperature: 60 °C; starch: 20%; AM: 15%; neutralization: 70%; MBA: 0.04%; and CAN and APS: 0.6% at a ratio of 1:3. The units of the above reaction factors were % *w*/*w*. The ratio of AA to AM was 4; the higher absorbency value was attributed to the transformation of grafted polymer AA into a poly (sodium acrylate), which was a highly ionized salt and hence, favored swelling [26].

### 2.3. Preparation of CST-PRP-SAP and Optimization of Reaction Conditions

Based on the synthesized CST-SAP samples, the PRP samples were mixed into the CST-SAP reaction system to synthesize the CST-PRP-SAP samples with the same graft copolymerization [27]. 

An orthogonal experiment of L_16_ (4^5^) was conducted, and the water absorbency and phosphorus release amount of CST-PRP-SAP samples were compared in the presence of different amounts of the PRP and AM, crosslinking agent, initiator, and neutralization degree (Table 1). The PRP contents were 2.18 g, 2.90 g, 3.63 g, and 4.36 g, which could convert to 7.5%, 10.0%, 12.5%, and 15.0% of P_2_O_5_ content. The reaction temperature of the system was 60℃, with the starch amount of 20% *w*/*w*. Based on the results of the orthogonal test, the effects of P_2_O_5_ contents (0%, 5%, 7.5%, 10%, and 12.5%) and neutralization degrees (50%, 60%, 70%, 80%, and 90%, with a P_2_O_5_ content of 10%) on the performances of water absorption and retention, and phosphorus release capacities of CST-PRP-SAP samples were all compared under the reaction conditions as follows: neutralization degree: 70%; reaction temperature: 60 ℃; starch content: 20%; AM content: 15%; crosslinking agent: 0.02%; and initiator 0.6%, respectively.

### 2.4. Characterization Techniques

#### 2.4.1. FTIR Analysis

The FTIR spectra were obtained using an FTIR spectrometer (Nicolet, Avatar 370, USA) to determine the functional groups of the CST, PRP, CST-SAP, and CST-PRP-SAP samples. All of the samples were compression-molded into a thin layer of files at 23 °C and about 3.0 MPa of pressure. The samples were cooled to room temperature (25 °C) in the mold under pressure and then scanned in the range of 400–4000 cm^−1^.

#### 2.4.2. XRD and Optical Microscope Analysis

The crystals and components of the CST, PRP, CST-SAP, and CST-PRP-SAP samples were analyzed using an XRD analysis (Bruker, AXS-D8-Focus, Freilassing, Germany) and transmission reflection polarizing microscope (Laica, DM2500P, Wetzlar, Germany). The CuKα ray was used as a radiation source, and the tube pressure and tube flow were 40 kV and 40 mA, respectively.

#### 2.4.3. Morphological Studies

The morphological characterizations of the CST, PRP, CST-SAP, and CST-PRP-SAP samples were made using a SEM analysis (FEI Hong Kong Hitachi Co., Quanta200, Hong Kong, China) at an acceleration of 25 kV. The samples were sputtered with a thin layer (~20 nm) of gold prior to SEM observation.

All the above-tested CST-SAP and CST-PRP-SAP samples were synthesized under the reaction conditions as follows: reaction temperature, starch content, crosslinking agent, initiator, neutralization degree, and AM content were 60 °C, 20%, 0.02%, 0.6%, 70%, and 15%, respectively, with a P_2_O_5_ content of 10%.

### 2.5. Index Measurement and Method

#### 2.5.1. Water Absorbency and Water Retention Behaviors Studies

A certain quality of the completely dried, pre-weighed CST-PRP-SAP samples were immersed in excess distilled water, tap water, and 0.9% NaCl solution, respectively, at room temperature (25 °C) to swell equilibrium. The swollen absorbent was taken out at regular intervals, the surface of the filter paper was wiped to remove the water bound on the surface, weighed, and then placed in the same bath. The quality measurement was continued until the equilibrium of the swelling behavior was reached. Water absorbency (*Q*) was determined using the following expression [27]:

*Q_e_* = (*m_e_* − *m*_0_)/*m*_0_ (1)

*Q_t_* = (*m_t_* − *m*_0_)/*m*_0_ (2)

where *m*_0_, *m_t_*, and *m_e_* represented the weights of the dry sample, swollen sample at time *t*, and balance, respectively (g). *Q_e_* and *Q_t_* were calculated as grams of water per gram of absorbent (g g^−1^). All the experiments were carried out five times for all samples and the average values were reported in this study. Water absorption rate or swelling rate could be reflected by the curve of water absorbency over time.

To measure the water retention of the modified samples, the completely swollen samples in distilled water were weighed, put in a weighing bottle, and placed into the constant temperature and humidity test chamber under the condition of 35% humidity at 40 °C. Then, the weight of each of the samples were measured at regular intervals. Water retention rate was calculated according to the following formula [24]:

*w_t_* = *m_t_*/*m*_0_ (3)

where *w_t_* denoted the water retention rate at time *t* (%). *m_t_* and *m*_0_ were the weights of dried sample after drying at time *t* and the water-absorbing saturated sample before drying, respectively (g).

To measure the repeated water absorbency of the modified samples, the completely swollen samples in distilled water were placed in a drying oven at a temperature of 70 °C to dry to a constant weight, and placed in distilled water again for repeated water absorption. Then, they were dried again for weighing after 48 h. Such drying and water absorption process were repeated. Repeated water absorbency was calculated using the following formula [28]:

*Q_n_* = (*m_n_*_−_
*m_n_*_0_)/*m_n_*_0_ (4)

where *Q_n_* represented the equilibrium water absorbency after each re-soaking (g g^−1^). *m_n_* was the weight of the sample at swelling equilibrium after each re-soaking (g). *m_n_*_0_ was the weight of the sample after each drying (g). Repeated water absorbency could be expressed by the curve of water absorbency over the dry–wet cycling time.

#### 2.5.2. Phosphorus Release Behaviors Tests

To study the slow release behaviors of different samples in distilled water, 0.5 g dried PRP and CST-PRP-SAP samples were added into conical bottles containing 250 mL distilled water. The bottles were put into incubators whose temperatures were set at 25 °C. At certain time intervals, a 5 mL solution was sampled for phosphorus determination, and an additional 5 mL of distilled water was injected into the bottles to maintain a constant amount of solvent. The phosphorus release capacity was estimated by an element analysis instrument [29].

*M* = (*m*/*v*) × *V* (5)

*W* = *M*/*M*_0_ (6)

where *M* and *W* denoted the cumulative phosphorus release amount (mg) and cumulative phosphorus release rate (%), respectively. *m* was the absorbance of the test solution that corresponded to the P_2_O_5_ content on the standard operating curve (mg). *v* was the volume of soaking liquid that was used for color reaction (mL). *V* was the volume of the sample soaking solution (mL). *M*_0_ was the total P_2_O_5_ content of the sample (mg).

#### 2.5.3. Kinetic Model and Sugihara Kinetic Model

In order to more clearly characterize the initial water absorption-swelling process of CST-PRP-SAP sample, the kinetic model was used to analyze the process [30].

(*m_t_* − *m*_0_)/(*m_max_* − *m*_0_) = *kt^n^* (7)

The logarithm on both sides of the equation could be obtained:

ln (*Q_t_*/*Q_max_*) *= n*ln*t* + ln*k* (8)

where *m_t_* and *m_max_* were the weights of SAP at time *t* and swelling equilibrium (g); *m*_0_ was the weight of dried SAP (g); *k* was the swelling rate constant of SAP; *t* was water absorption time (min); *n* was the characteristic index of water absorption-swelling of SAP, indicating the type of reaction-diffusion, and its value described the control process of the water absorption rate. *Q_t_* and *Q_max_* were the water absorbency of SAP at time *t* and swelling equilibrium (g g^−1^). 

When *n* ≤ 0.5, it conformed to Fickian diffusion behavior, and the diffusion rate of water molecules into the SAPs network was very slow, which played a decisive role in the swelling process; when 0.5 < *n*< 1, there was non-Fickian diffusion or irregular diffusion, which was determined by water diffusion and polymer chain relaxation; when *n* ≥ 1, non-Fickian diffusion occurred with a fast diffusion rate of water molecules, and a small relaxation rate of polymer chain segments, so the whole process was determined by the latter.

To clarify the mechanism of influences of PRP contents on the cumulative phosphorus release amount and cumulative phosphorus release rate of CST-PRP-SAP, the Sugihara kinetic model was used for curve fitting [31].

*m* = *a*(1 − *e*^ − *kt*^) (9)

*w* = *b*(1 − *e*^ − *kt*^) (10)

where *t* was soaking time (h); *m* and *w* were the cumulative phosphorus release amount (mg) and cumulative phosphorus release rate (%) at time *t*; Constant *a* was the phosphorus amount activated by AA through acid dissolution during polymerization at 60 °C (mg), indicating the activation capacity of AA in the system for insoluble phosphorus in PRP; Constant *b* was the phosphorus rate activated by AA through acid dissolution (%), indicating the activation degree of AA in the system to insoluble phosphorus in PRP; Constant *k* was the phosphorus release rate during the extraction process, indicating the change rate of the cumulative release amount with time, and representing the contribution of SAPs network system to the phosphorus release.

## 3. Results and Discussion

### 3.1. Valence Bond Structure and Morphological Characteristics

#### 3.1.1. FTIR Spectra Analysis

**Figure 1:**
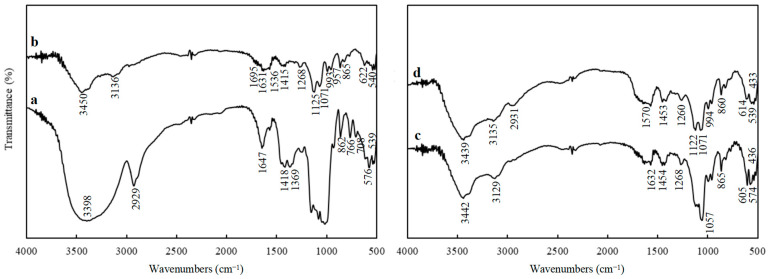
The Fourier transform infrared spectroscopy of the cassava starch (CST, (**a**)), cassava starch-based super-absorbent polymer (CST-SAP, (**b**)), powdered rock phosphate (PRP, (**c**)), and cassava starch-based phosphorus releasing super-absorbent polymer (CST-PRP-SAP, (**d**)).

By comparing and analyzing the absorption peaks of functional groups in the infrared spectrum of CST and CST-SAP samples, the grafting copolymerization with AA could be judged [32]. In the FTIR of CST, the bands at 862 cm^−1^, 766 cm^−1^, 708 cm^−1^, 576 cm^−1^, and 539 cm^−1^ were characteristic absorption peaks of starch, the bands at 1155 cm^−1^, 1081 cm^−1^, and 1018 cm^−1^ were asymmetric stretching vibration absorption peaks of starch C-O-C, the bands at 2929 cm^−1^ were saturated C-H stretching vibration absorption peaks, and the broad bands at 3398 cm^−1^ were -OH stretching vibration absorption peaks on starch molecules (Figure 1). The characteristic absorption peaks of starch at 993 cm^−1^, 957 cm^−1^, 865 cm^−1^, 622 cm^−1^, and 540 cm^−1^ were retained in the infrared spectrum of the grafted product CST-SAP. The C-O-C stretching vibration absorption peaks of starch at 1125 cm^−1^ and 1071 cm^−1^ were significantly weakened, and the starch-OH stretching vibration absorption peaks at 3450 cm^−1^ were also narrowed and weakened, indicating that the starch had undergone grafting copolymerization. The band generated at 3136 cm^−1^ was the -CH stretching vibration absorption peak of the -[CH_2_-CH]- unit in the structure of the graft copolymerization product, which further proved occurrence of the graft copolymerization reaction. The maintained -OH and -CH vibration absorption peaks were the important bands for both starch and AA moieties in hydrogel [33]. The band at 1695 cm^−1^ and 1631 cm^−1^ was the stretching vibration absorption peak of C=O under the combined influence of acrylic acid -COOH and acrylamide -CONH_2_. The band at 1536 cm^−1^ was the bending vibration absorption peak of -NH_2_. The band at 1415 cm^−1^ was the symmetric stretching vibration absorption peak of -COO, but the absorption intensity was weak due to its partial neutralization, while the band at 1268 cm^−1^ was the C-N stretching vibration absorption peak, indicating that the crosslinking agent *N*,*N*-MBA participated in the reaction and played a cross-linking role. This showed that CST has graft copolymerization with AA and AM. 

The spectral bands of PRP samples at approximately 3442 cm^−1^, 3129 cm^−1^, and 1632 cm^−1^ were the stretching vibration absorption peaks of adsorbed water, and the spectral bands at approximately 1454 cm^−1^ and 1268 cm^−1^ were the antisymmetric stretching vibration absorption peaks of CO_3_^2−^ (Figure 1). The strong band at approximately 1057 cm^−1^ was the antisymmetric stretching vibration absorption peak of PO_4_^3−^, the bands at wavelength 865 cm^−1^, 605 cm^−1^, and 574 cm^−1^ were the antisymmetric bending vibration absorption peaks of PO_4_^3−^, and that at wavelength 436 cm^−1^ was the symmetric bending vibration absorption peak of PO_4_^3−^, indicating that the insoluble phosphorus in the PRP sample mainly existed in the form of phosphate. 

The CST-PRP-SAP sample had a stretching vibration absorption peak which represented free -OH at approximately 3439 cm^−1^, a stretching vibration absorption peak represented -CH of graft copolymerization unit -[CH_2_-CH]- at approximately 3135 cm^−1^, a characteristic absorption band related to PO_4_^3−^ at approximately 2931 cm^−1^, and -NH_2_ bending vibration absorption peak, -COO symmetric stretching vibration absorption peak, and C-N stretching vibration absorption peak at approximately 1570 cm^−1^, 1453 cm^−1^, and 1260 cm^−1^, respectively, which directly proved the existence of a cross-linked SAPs network structure. The spectral band at approximately 1122 cm^−1^ was the weakened C-O-C stretching vibration absorption peak of starch, and the antisymmetric stretching vibration absorption peak of PO_4_^3−^ in PRP at approximately 1071 cm^−1^ was also weakened. The series of spectral bands at approximately 994 cm^−1^, 860 cm^−1^, 614 cm^−1^, and 539 cm^−1^ were the joint influence of starch characteristic absorption and the antisymmetric bending vibration absorption of PO_4_^3−^ in PRP, and the wave length at approximately 433 cm^−1^ was the symmetric bending vibration absorption peak of PO_4_^3−^. The results showed that the CST-PRP-SAP was the product of graft copolymerization, and most of the PRP existed in the system in the form of physical filling, and some fraction of the PRP was activated to release phosphorus.

#### 3.1.2. XRD Analysis

**Figure 2:**
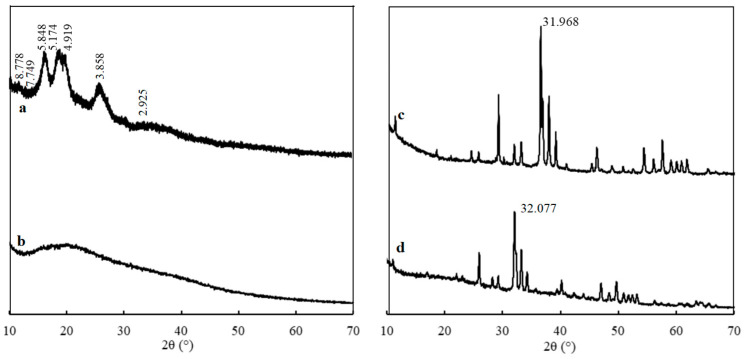
The X-ray diffraction pattern of the cassava starch (CST, (**a**)), cassava starch-based super-absorbent polymer (CST-SAP, (**b**)), powdered rock phosphate (PRP, (**c**)), and cassava starch-based phosphorus releasing super-absorbent polymer (CST-PRP-SAP, (**d**)).

By comparing the XRD patterns of the CST and CST-SAP samples, it was found that the characteristic diffraction peaks of the CST appeared at 2θ = 10.0°, 11.4°, 15.1°, 17.1°, 18.0°, 23.0°, and 30.5°, indicating that the starch had a certain crystalline structure (Figure 2). However, these characteristic diffraction peaks did not appear in the synthesized CST-SAP, nor even any other new diffraction peaks. The entire XRD pattern of CST-SAP showed a smooth hill-shaped shape, namely a “steamed bread peak”, which showed that the copolymerization destroyed the original crystalline structure of CST, and the graft product was an amorphous structure. It might be more conducive to the degradation of CST-SAP under natural conditions.

The PRP sample at 2θ = 31.968° represented the apatite crystallization characteristics of the main peak shape sharp with high crystallinity (Figure 2). The basic characteristic diffraction peaks of the PRP were preserved in the CST-PRP-SAP samples, and the main characteristic diffraction peaks were passivated at the corresponding 2θ = 32.077°, indicating that the crystallization degree of PRP in the reaction system was reduced. The available phosphorus content in PRP was inversely proportional to the main peak height, showing that the available phosphorus content in the CST-PRP-SAP had increased to a certain extent, and part of the insoluble phosphorus in the PRP sample could be converted into available phosphorus.

#### 3.1.3. SEM Analysis

**Figure 3:**
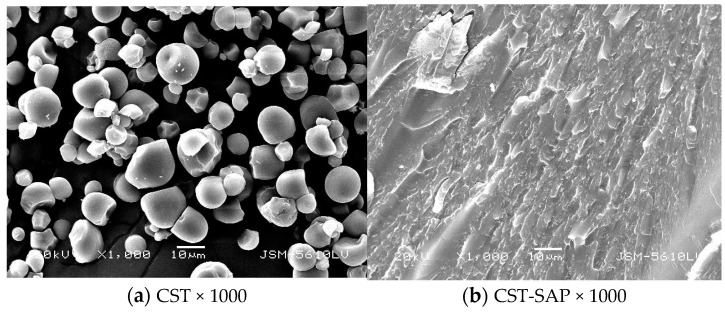

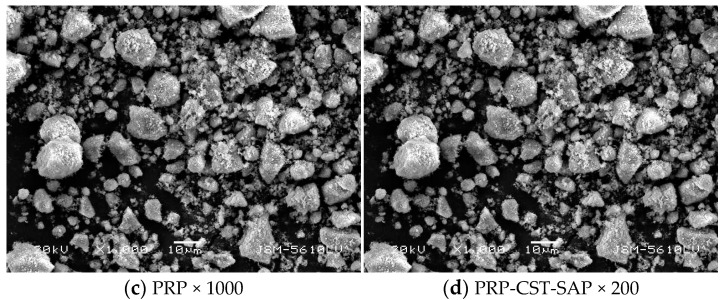
The scanning electron microscopy images of the cassava starch (CST, (**a**)), cassava starch-based super-absorbent polymer (CST-SAP, (**b**)), powdered rock phosphate (PRP, (**c**)), and cassava starch-based phosphorus releasing super-absorbent polymer (CST-PRP-SAP, (**d**)). The magnifications of ×1000 and ×200 in the samples were adjusted appropriately to obtain clear images due to the differences in morphology and thickness of the samples.

The SEM visualizations of different CST, PRP, and dried CST-SAP and CST-PRP-SAP samples were shown in Figure 3. The CST particles were spherical or semi-spherical with a smooth and closely arranged surface (Figure 3a). The surface of CST-SAP was extremely rough and had a layered structure full of folds, which was conducive to increasing the specific surface area of the material and facilitating the diffusion of water molecules from the surface into the network (Figure 3b). The particle size distribution of the PRP sample was uneven, the appearance was gray white, and the particles were short columns or irregular thick plates (Figure 3c). While the surface of the CST-PRP-SAP sample was relatively rough and distributed in folds (Figure 3d), which was conducive to increasing the specific surface area of the CST-PRP-SAP, and promoting its water absorption, its functional properties of water absorption and retention were somewhat similar to the reported nanocomposite coated fertilizer [34]. Meanwhile, part of the PRP particles were also wrapped in the CST-PRP-SAP surface. This might be related to the fact that a large amount of PRP was not fully and evenly mixed in the reaction system through mechanical stirring, and a small amount of agglomeration occurred, which would affect the PRP activation of the reaction system to a certain extent.

#### 3.1.4. Optical Microscope Analysis

**Figure 4:**
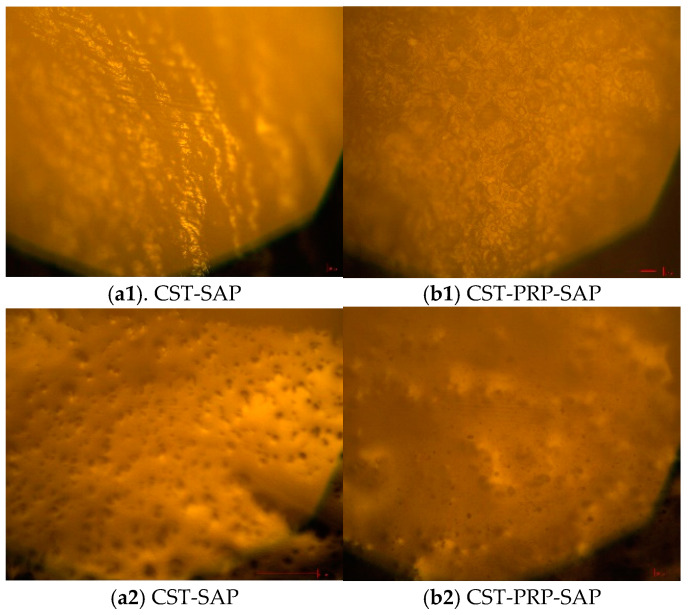
The optical micrographs of the cassava starch-based super-absorbent polymer (CST-SAP) and cassava starch-based phosphorus releasing super-absorbent polymer (CST-PRP-SAP) before water absorption (**a1**,**b1**) and after water absorption (**a2**,**b2**).

The surface of the dried CST-SAP sample before water absorption was dense and rough with layered wrinkles (Figure 4(a1)), while it expanded into a translucent gel after water absorption (Figure 4(a2)). The micro-holes on the surface had a strong water absorption capacity, which might play a role in improving the water absorption performance of the material. The particle surface of the dried CST-PRP-SAP sample before water absorption was rough and uneven (Figure 4(b1)). While it was in the form of translucent gel after water absorption and expansion, the water was kept in the gel network, and the PRP was almost evenly distributed in the gel system (Figure 4(b2)). The microspores on the surface of the CST-PRP-SAP samples were also helpful to improve the water absorption and phosphorus release performance.

### 3.2. Effects of Different Reaction Conditions on Absorption and Phosphorus Release Capacities of the CST-PRP-SAP Samples

SAP had strong environmental adaptability, which helps to alleviate the adverse effects of drought stress on crops. In a practical application, the fertilizer in the soil would reduce the ion concentration difference between SAPs and the fertilizer solution, and weaken the absorption performance of SAPs [35]. In this study, based on the optimization and synthesis of CST-SAP, the abundant mineral resource of PRP was used as the main material, and a compound functional CST-PRP-SAP was prepared through aqueous solution polymerization. The water retention and phosphorus release performance of CST-PRP-SAP were optimized to meet the dual needs of soil water conservation and phosphorus release improvement. 

Table 1 showed the effects of the P_2_O_5_ and AM content, crosslinking agent, initiator, and neutralization on water absorbency and phosphorus release capacities of the CST-PRP-SAP sample. Through an orthogonal test, it was found that factors affect the water absorbencies in distilled water, tap water, and 0.9% NaCl solution as follows: crosslinking agent > P_2_O_5_ content > initiator > neutralization degree > AM content; P_2_O_5_ content > neutralization degree > crosslinking agent > AM content > initiator; P_2_O_5_ content > crosslinking agent > neutralization degree > initiator > AM content, respectively. The water absorbency of SAPs in tap water and the 0.9% NaCl solution was more valuable for reference in agriculture. Thus, the PRP content might be one of the most important factors affecting the water absorbent capacity of CST-PRP-SAP. The optimal level analysis in the orthogonal test further showed that the optimal conditions for maximum water absorbencies of the CST-PRP-SAP samples in different solutions were slightly different (Table 1). Those in distilled water were as follows: P_2_O_5_ content, crosslinking agent, initiator, neutralization degree, and AM content were 10%, 0.02%, 0.6%, 90%, and 15%, respectively. Yet, the optimal conditions were changed in tap water and the 0.9% NaCl solution; the AM content and initiator were reduced to 10% and 0.4%, respectively. 

The influence of different factors on the phosphorus release amount of CST-PRP-SAP was in the order of neutralization degree > P_2_O_5_ content > AM content > initiator > crosslinking agent, which indicated that the PRP content and neutralization degree were the main influencing factors. The results of the orthogonal test showed that the optimum conditions for the phosphorus release amount of CST-PRP-SAP in 7 d were as follows: P_2_O_5_ content, crosslinking agent, initiator, neutralization degree, and AM content were 15%, 0.08%, 0.6%, 60%, and 15%, respectively. Under such an optimum condition test, the release amount reached 8.69 mg in 7 d. Further combined with the results of water absorbencies, the optimum reaction conditions were obtained as follows: P_2_O_5_ content, crosslinking agent, initiator, neutralization degree, and acrylamide were 10%, 0.02%, 0.6%, 70%, and 15%, respectively. 

### 3.3. Effects of PRP Contents on the Water Absorption Capacities of the CST-PRP-SAP Samples

**Figure 5:**
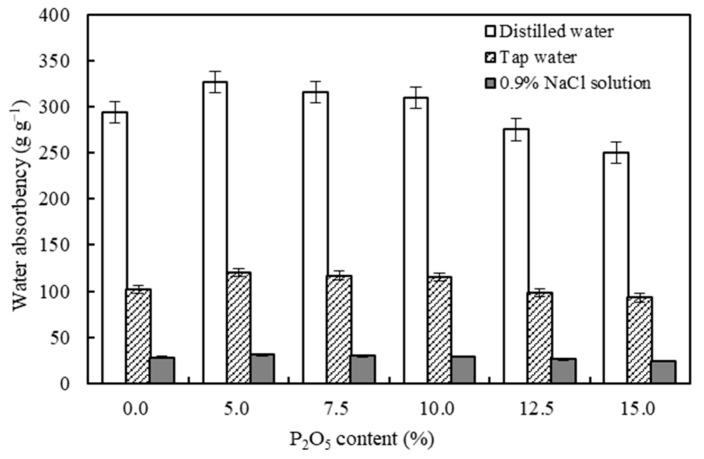
Effects of P_2_O_5_ contents on the water absorbency of the cassava starch-based phosphorus releasing super-absorbent polymer in different solutions.

Water absorbency was an important index to judge water absorption performance of SAPs [36]. In this study, the added PRP content was one of the most important factors affecting the liquid absorption performance of CST-PRP-SAP. Under the reaction conditions as follows, reaction temperature, crosslinking agent, initiator, neutralization degree, and AM content were 60 °C, 0.02%, 0.6%, 70%, and 15% respectively. Compared with the CST-SAP samples without adding PRP, the maximum water absorbencies of CST-PRP-SAP were about 327 g g^−1^, 120 g g^−1^, and 31 g g^−1^ in distilled water, tap water, and the 0.9%NaCl solution with a P_2_O_5_ content of 5%, and they were all significantly higher than those of the CST-SAP sample until the P_2_O_5_ content increased to 10% (Figure 5). When the PRP contents continued to increase, the water absorbency of CST-PRP-SAP would decline, even less than that of CST-SAP. The main reason might be that AA in the reaction system had a certain activation effect on relatively small amounts of PRP when polymerized at 60 °C, so that a small amount of insoluble phosphorus could be converted into ionic soluble phosphorus, and Ca^2+^ combined with phosphate could be released. It might increase the ion concentration in the SAP network and strengthen the role of osmotic pressure in the water absorption process. Meanwhile, a small amount of Ca^2+^ could also act as a cross-linking agent, which might improve the cross-linking density of CST-PRP-SAP to a certain extent, and enhance its liquid absorption performance. However, the reduction of water absorbency in the presence of more PRP might be due to the generation of more crosslinking points which restricted movement of the polymer chains, as the same effect reported on zeolite [37]. Most of the PRP could only play the role of filling composite, which would hinder the network space expansion of CST-PRP-SAP, and weaken its liquid absorption capacity.

**Figure 6:**
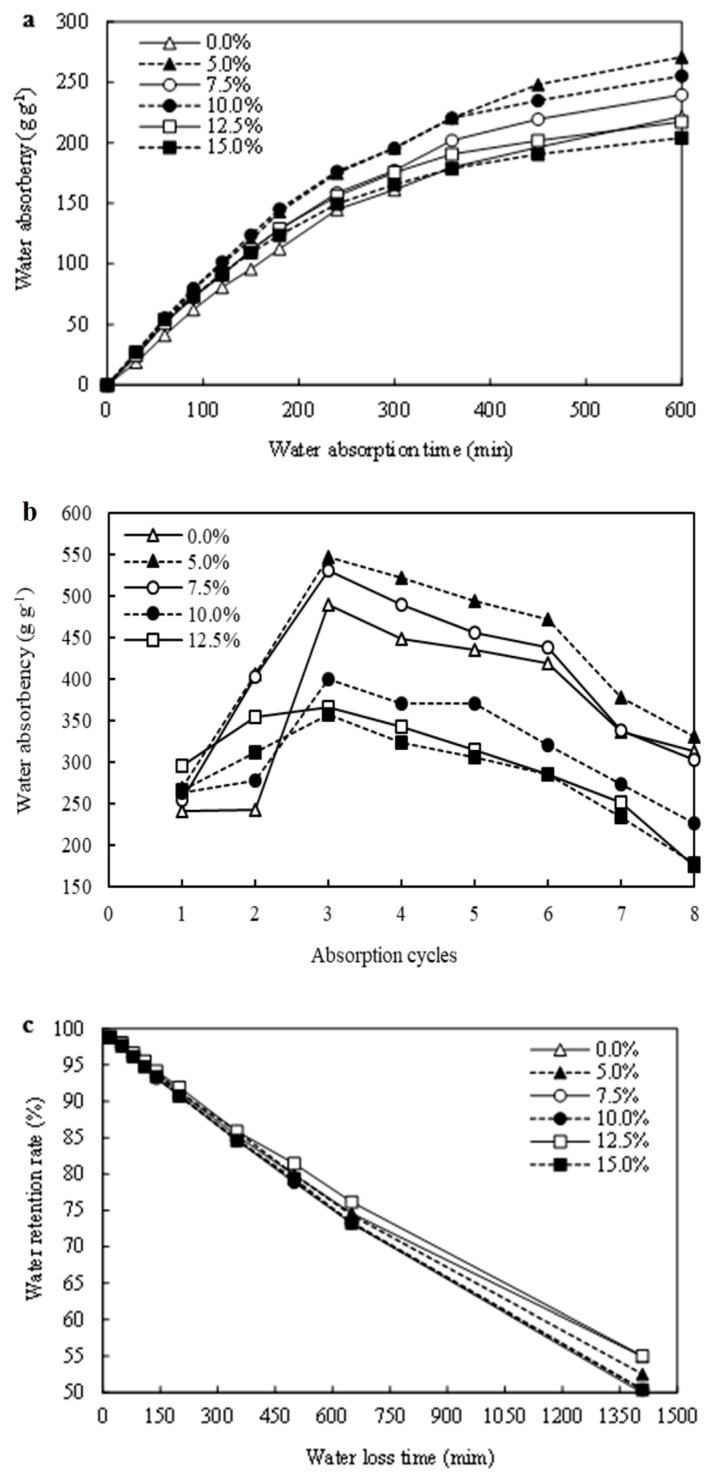
Effects of P_2_O_5_ contents on the characteristics of water absorbency (**a**), repeated water absorbency (**b**), and water retention rate (**c**) of the cassava starch-based phosphorus releasing super-absorbent polymer.

The water absorbency of CST-PRP-SAP showed the same change trend under different PRP contents (Figure 6a). It absorbed water rapidly in the first 180 min, and then the water absorbency slowed down gradually with the extension of absorption time. It was observed that the water absorbency of most treatments was significantly higher than that of the CST-SAP sample, except for some specific water absorption times with a P_2_O_5_ content of 12.5% and 15.0%, indicating that the appropriate adding of PRP could significantly improve the water absorbency of CST-PRP-SAP.

The reusability of SAPs could directly reflect the continuous effect of SAPs in practical applications, and was also the standard to measure whether it can be used for a long time [28]. It was necessary to coordinate the contradiction between the cross-linking degree and water absorption performance to ensure good performance of SAPs [32]. The maximum water absorbencies of the CST-SAP and CST-PRP-SAP sample were reached at the third time of water swelling (Figure 6b). After the third absorption cycles, the water absorbency of CST-PRP-SAP gradually decreased, and they were almost larger than those of CST-SAP with a P_2_O_5_ content of 5.0% and 7.5%. When the P_2_O_5_ contents were higher than 10%, the maximum equilibrium water absorbencies were smaller than those of CST-SAP samples. This might be related to homopolymerization accompanied in the process of polymerization, and homopolymerization products were filled in SAPs network as water-soluble oligomers, without forming an effective cross-linking network. 

The water holding capacity of SAPs referred to its internal hydrophilic structure and the strength of the water molecule interaction, that is, its ability to keep water from being separated [38]. The water retention capacity of SAPs was determined by its water absorption and water loss characteristics [1]. Under a drying condition of 40 °C, the water retention curves of each CST-PRP-SAP treatment showed the same trends with the increasing of P_2_O_5_ content, and they were consistent with the CST-SAP sample, especially in the first 400 min, as all curves were basically coincided (Figure 6c). It could be seen that the PRP content had no significant effect on the water retention performance of CST-PRP-SAP. The rapid water loss stage was observed within 200 min of the initial drying, and about 10% of the initial water content was reduced. With the extension of water loss time, the water retention rate gradually slowed down with a decreasing trend. The CST-PRP-SAP could still maintain more than 50% of the initial moisture after 1400 min, indicating that they could retain a high water content for a long time even in a continuously higher ambient temperature.

### 3.4. Water Absorption-Swelling Process of the CST-PRP-SAP Samples

The water absorption-swelling process of SAP was generally as follows: firstly, water molecules diffused to the interior of SAPs through the action of hydrophilic groups on the surface of SAPs. The concentration difference between the internal and external structures of the network was caused by diffusion, which lead to the opening of the polymer network. Finally, the polymer network structure expanded to make the water absorption of SAPs reach equilibrium [39]. If the water absorption rate depended on the diffusion rate of water molecules, the liquid absorption amount of SAP was proportional to the square root of time [40]. If the water absorption rate depended on the expansion rate of the polymer chain segment, the absorbent of SAPs was proportional to the water absorption time, and the square root of water absorption time into an S-shaped change [39]. 

The value of *n* with different PRP contents was between 0.6 and 0.9 (Table 2), indicating that the water absorption-swelling process of the tested CST-PRP-SAP sample conformed to non-Fickian diffusion, which was determined by both the diffusion of water molecules and relaxation process of polymer chain segments. With the increase of PRP content, *n* decreased in turn, while the swelling rate constant *k* showed an increasing trend, in which the absorption-swelling rate of CST-PRP-SAP would increase with the increase of PRP content. In addition, adding PRP not only increased the diffusion of water molecules, but also effectively reduced the relaxation rate of polymer chains. This might be due to the increase of ion concentration difference inside and outside of the system after PRP was activated in a small amount in the reaction system, thus accelerating the diffusion rate of water molecules. The activated free ions gradually diffused from the inside of the polymer network to the outside, which increased the osmotic pressure of the external solution, inhibited the further extension of polymer chain, and thus reduced the relaxation rate of the polymer chain.

### 3.5. Effects of PRP Contents on the Phosphorus Release Capacity of the CST-PRP-SAP Samples

As a composite functional water retaining material, it should improve the soil water status, increase soil nutrients absorption, reduce leaching loss, and improve the crop’s absorption of nutrients loaded by SAPs [41]. Previous studies have confirmed that while the SAPs carried out molecular bonding and swelling absorption, the nutrient ions in the solution would also enter its molecular structure and be wrapped and fixed, and then be slowly released with the release of water and the relaxation of the molecular network structure, playing a slow release effect of nutrient ions [42].

**Figure 7:**
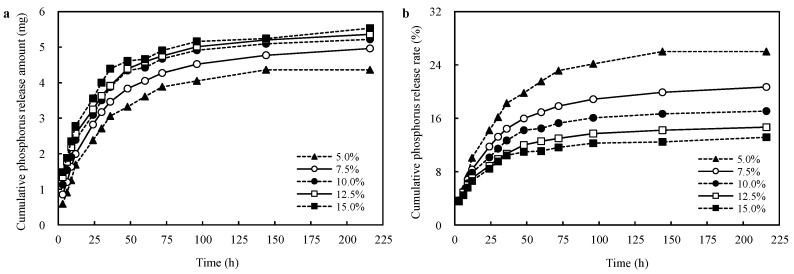
Effects of P_2_O_5_ contents on the cumulative phosphorus release amount (**a**) and cumulative phosphorus release rate (**b**) of the cassava starch-based phosphorus releasing super-absorbent polymer.

The influences of different neutralization degrees on the phosphorus release capacities of CST-PRP-SAP samples were compared under the reaction condition, neutralization degree: 70%; reaction temperature: 60 °C; starch content: 20%; AM content: 15%; crosslinking agent: 0.02%; and initiator 0.6%, respectively, with a P_2_O_5_ content of 10%. Different PRP contents conducted consistent impact trends on the phosphorus release amount and release rate, and they all increased gradually with the extension of extraction time; especially in the initial 24 h, they all increased sharply. The rising trend was gradually flat within 24~96 h, and then released slowly and became stable after 96~216 h (Figure 7). The cumulative phosphorus release amount of CST-PRP-SAP increased with the increasing PRP content, while the phosphorus release rate decreased gradually. The results of the water extraction experiment showed that after 226 h of leaching, the phosphorus release amount and release rate of CST-PRP-SAP samples with different PRP contents increased by 17.4 and 3.7 times of the initial; especially in the first 24 h, the maximum phosphorus release amount was obtained. It could be seen that CST-PRP-SAP had a slow release and accelerated release effect on phosphorus.

The constants *a* and *k* were gradually increased, and *b* was decreased with the increasing PRP content (Table 3). The results indicated that the activation ability of insoluble phosphorus and the phosphorus release rate during soaking could be improved to a certain extent by increasing PRP content, but the rate of phosphorus activated by AA through acid dissolution could be inhibited. This phenomenon might occur when AA amounts remain unchanged, the PRP density in the system was increased with the increasing PRP content, and the contact probability between PRP and AA would also be improved during polymerization, thus increasing the activation of AA on PRP, so the value *a* would increase accordingly.

The phosphorus release rate was the ratio of phosphorus release amount to the total P_2_O_5_ content in the system. Although AA had a certain acid dissolution activation effect on insoluble phosphorus in PRP, this effect was limited under the condition of a certain amount of AA. The total P_2_O_5_ content in the system was increased with the increasing PRP content, and the proportion of PRP that could not be activated by AA also increased rapidly. The un-activated PRP accounted for the vast majority, and the growth rate of its proportion was greater than the phosphorus amount released by a small part of PRP that could be activated by AA, so the value *b* gradually decreased. In the process of extraction, the water-soluble ionic phosphorus released by the activated part of PRP in the SAPs network increased with the increasing PRP content, leading to the increase of ion concentration in the network. This soluble phosphorus would move from the inside of the SAPs network to the external aqueous solution under the action of osmotic pressure, and the trend was more obvious with the larger ion concentration difference. Therefore, the increasing rate of the cumulative phosphorus release amount during the extraction process was faster, so the value *k* was larger. The dissolved phosphorus would gradually increase the osmotic pressure of the external aqueous solution of the SAPs network, thus inhibiting the further extension of the polymer chain and hindering the expansion of the SAPs network space, which would reduce the adsorption and retention of phosphorus in the SAPs network, and promote the increase of value *k* to a certain extent.

In an actual farmland application, the PRP content could be adjusted according to the top priority. Generally, the synthetic CST-PRP-SAPs with a P_2_O_5_ content of 10% had functions of both water absorption and phosphorus slow-release. If the CST-PRP-SAPs would be applied to farmland with a high temperature or frequent droughts, the recommended P_2_O_5_ content was 5% for improving the soil moisture status as the primary goal. When they were used on phosphorus-loving crops or dry soil with low phosphorus and phosphorus deficiency, it was suggested that the P_2_O_5_ content could be increased to 15% to obtain a larger phosphorus release ability.

### 3.6. Effects of Different Neutralization Degrees on the Phosphorus Release Capacity of the CST-PRP-SAP Samples

**Figure 8:**
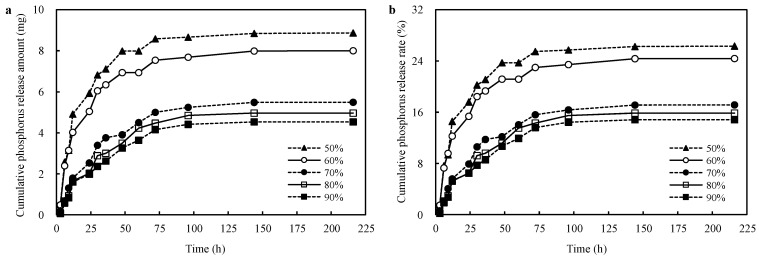
Effects of neutralization degrees on the cumulative phosphorus release amount (**a**) and cumulative phosphorus release rate (**b**) of the cassava starch-based phosphorus releasing super-absorbent polymer.

The cumulative phosphorus release amount and release rate were all increased gradually with the extension of extraction time; especially in the initial 24 h, they rose sharply and the rising trend was gradually flat within 24~96 h. Then, the increment was stable, and the variation was not obvious after 96~216 h (Figure 8). The cumulative phosphorus release amount and release rate of CST-PRP-SAP also showed a significant downward trend with the increasing of the neutralization degree, that is, the phosphorus release characteristics were negatively related to the neutralization degree.

Further, the Sugihara kinetic model was used to fit the curves of the cumulative phosphorus release amount and release rate of samples with different neutralization degrees. It was found that the constants *a*, *b*, and *k* decreased with the increase of the neutralization degree (Table 4), that is, the phosphorus amount and phosphorus rate activated by AA dissolution during polymerization at 60 °C, as well as the phosphorus release rate during extraction, were negatively related to the neutralization degree, indicating that a relatively lower neutralization degree was more conducive to improving the phosphorus release performance of CST-PRP-SAP, which was consistent with the orthogonal test results (Table 1). This might be due to the relative increase of -COOH content of AA in the system with the addition of a certain amount of PRP, when the neutralization degree decreased. That is, the higher concentration of -COOH, the more H^+^ would be dissociated during polymerization, which would improve the AA ability to activate PRP through acid dissolution during reaction. On the contrary, with the increase of the neutralization degree, the content of H^+^ in the system decreased, and this acid solution activation was also weakened, resulting in the decrease of *a*. The total P_2_O_5_ content in the system remained unchanged with fixed PRP content, and the phosphorus release rate was proportional to the phosphorus release amount, so value *b* decreased accordingly. During the extraction process, with the increase of the neutralization degree, the ionic phosphorus activated inside the SAPs network decreased, reduced the ion concentration difference inside and outside the network, and promoted the osmotic pressure of phosphorus dissolved from the inside of the SAPs network to the outside aqueous solution which also decreased. Meanwhile, the amount of -COONa in the system with a higher dissociation degree than -COOH was relatively increased, which increased the concentration of -COO^−^, improved the electrostatic repulsion between -COO^−^ negative charges on the polymer chain of network, and promoted the extension of the polymer chain and expansion of the network space, thereby increasing the adsorption and retention of phosphorus by the SAPs network. Under the combined influence of the above factors, the phosphorus release rate and value *k* decreased with the increase of the neutralization degree.

## 4. Conclusions

In this work, we aimed at the synthesis of a novel CST-PRP-SAP, for the effective use of starch and rock phosphate powder; the properties of the CST, PRP, CST-SAP, and CST-PRP-SAP samples were all compared by using morphologic and structural characterization. The optimized behaviors of water absorption and phosphorus release were conducted through experimental methods. The following conclusions were drawn: (1) the CST-PRP-SAP was the product of graft copolymerization, and most of the PRP existed in the system in the form of physical filling. The PRP was almost evenly distributed in the CST-PRP-SAP samples, and its micro-pore structure on the surface after water absorption helped to improve the performance of water retention and phosphorus release; (2) under optimized process conditions, the synthesized CST-PRP-SAP had functions of water absorption and retention, phosphorus slow-release, and good reusable performance; (3) the PRP content was the most important factors affecting the water absorbency of CST-PRP-SAP, while the neutralization degree was the key factor for the phosphorus release performance.

## Figures and Tables

**Table 1:** Orthogonal experimental results for water absorbency and phosphorus release amount of cassava starch-based phosphorus releasing super-absorbent polymer.

Number	Reaction Factors ^a^ (% *w*/*w*)					Water Absorbency (g g^−1^)			Phosphorus Release Amount (mg)
	A	B	C	D	E	Distilled Water	Tap Water	0.9% NaCl Solution	
1	7.5	0.02	0.4	60	10	204	76	23	5.87
2	7.5	0.04	0.6	70	15	225	80	22	6.04
3	7.5	0.06	0.8	80	20	128	71	21	5.22
4	7.5	0.08	1.0	90	25	127	67	20	4.41
5	10.0	0.02	0.6	80	25	275	102	27	5.11
6	10.0	0.04	0.4	90	20	264	95	29	4.94
7	10.0	0.06	1.0	60	15	186	70	22	6.25
8	10.0	0.08	0.8	70	10	145	83	21	5.98
9	12.5	0.02	0.8	90	15	204	86	22	5.29
10	12.5	0.04	1.0	80	10	170	71	21	5.80
11	12.5	0.06	0.4	70	25	121	55	18	5.91
12	12.5	0.08	0.6	60	20	131	51	17	6.76
13	15.0	0.02	1.0	70	20	160	67	18	6.18
14	15.0	0.04	0.8	60	25	126	46	16	6.03
15	15.0	0.06	0.6	90	10	174	84	20	5.51
16	15.0	0.08	0.4	80	15	135	61	17	6.08
Mean 1	171.00	210.75	181.00	161.75	173.25	Water absorbency in distilled water (g g^−1^)			
Mean 2	217.50	196.25	201.25	172.75	187.50				
Mean 3	156.50	152.25	150.75	177.00	170.75				
Mean 4	148.75	134.50	160.75	192.25	162.25				
Range	68.75	76.25	50.50	30.50	25.25				
Primary and secondary factors				B > A > C > D > E					
Optimal level				A_2_B_1_C_2_D_4_E_2_					
Mean 1	73.50	82.75	71.75	60.75	78.50	Water absorbency in tap water (g g^−1^)			
Mean 2	87.50	73.00	79.25	71.25	74.25				
Mean 3	65.75	70.00	71.50	76.25	71.00				
Mean 4	64.50	65.50	68.75	83.00	67.50				
Range	23.00	17.25	10.50	22.25	11.00				
Primary and secondary factors				A > D > B > E > C					
Optimal level				A_2_B_1_C_2_D_4_E_1_					
Mean 1	21.50	22.50	21.75	19.50	21.25	Water absorbency in 0.9% NaCl solution (g g^−1^)			
Mean 2	24.75	22.00	21.50	19.75	20.75				
Mean 3	19.50	20.25	20.00	21.50	21.25				
Mean 4	17.75	18.75	20.25	22.75	20.25				
Range	7.00	3.75	1.75	3.25	1.00				
Primary and secondary factors				A > B > D > C > E					
Optimal level				A_2_B_1_C_1_D_4_E_1_					
Mean 1	5.39	5.61	5.70	6.23	5.79	Phosphorus release amount (mg)			
Mean 2	5.57	5.70	5.86	6.03	5.92				
Mean 3	5.94	5.72	5.63	5.55	5.78				
Mean 4	5.95	5.81	5.66	5.04	5.37				
Range	0.57	0.20	0.23	1.19	0.55				
Primary and secondary factors				D > A > E > C > B					
Optimal level				A_4_B_4_C_2_D_1_E_2_					

^a^ Reaction factions of A, B, C, D, and E referred to P_2_O_5_ content, crosslinking agent, initiator, neutralization degree, and acrylic amide content, respectively.

**Table 2:** The model fitting parameters of the experimental samples with different P_2_O_5_ contents.

P_2_O_5_ Content (%)	Linear Fitting Equation ^a^	*n* ^b^	*Lnk* ^c^	*R*^2^ ^d^
0.0	*y* = 0.823*x* − 5.052	0.823	−5.052	0.970
5.0	*y* = 0.806*x* − 4.943	0.806	−4.943	0.972
7.5	*y* = 0.770*x* − 4.724	0.770	−4.724	0.972
10.0	*y* = 0.760*x* − 4.637	0.760	−4.637	0.966
12.5	*y* = 0.729*x* − 4.430	0.729	−4.430	0.961
15.0	*y* = 0.675*x* − 4.107	0.675	−4.107	0.961

^a^ from the kinetic model, and obtained by ln*t* as the *x*, and ln(*Q_t_*/*Q_max_*) as the *y*; ^b^ was the characteristic index of water absorption-swelling; ^c^ referred to the natural logarithm of swelling rate constant; ^d^ was the coefficient of determination.

**Table 3:** The Sugihara model fitting parameters of the experimental samples with different P_2_O_5_ contents.

P_2_O_5_ Content (%).	*a* ^a^	*b* ^b^	*k* ^c^	*R* ^2 d^
5.0	4.259	25.394	0.035	0.991
7.5	4.666	19.465	0.040	0.980
10.0	4.932	16.141	0.047	0.965
12.5	4.971	13.596	0.052	0.937
15.0	5.051	12.003	0.062	0.943

^a^ was the phosphorus amount activated by AA through acid dissolution; ^b^ was the phosphorus rate activated by AA through acid dissolution; ^c^ referred to the phosphorus release rate during the extraction process; ^d^ was the coefficient of determination.

**Table 4:** The Sugihara model fitting parameters of the experimental samples with different neutralization degrees.

Neutralization Degree (%)	*a*	*b*	*k*	*R* ^2^
50	8.702	25.823	0.052	0.980
60	7.740	23.590	0.050	0.979
70	5.562	17.365	0.029	0.991
80	5.139	16.429	0.026	0.986
90	4.698	15.373	0.025	0.985

All constants in this table have the same meaning as those in Table 3.

## Data Availability

The data used to support the findings of this study are available from the corresponding author upon request.
